# Increasing Prevalence of *Borrelia burgdorferi* sensu stricto–Infected Blacklegged Ticks in Tennessee Valley, Tennessee, USA

**DOI:** 10.3201/eid2409.180343

**Published:** 2018-09

**Authors:** Graham J. Hickling, Janetta R. Kelly, Lisa D. Auckland, Sarah A. Hamer

**Affiliations:** University of Tennessee Institute of Agriculture, Knoxville, Tennessee, USA (G.J. Hickling, J.R. Kelly);; Texas A&M University, College Station, Texas, USA (L.D. Auckland, S.A. Hamer)

**Keywords:** *Ixodes scapularis*, *Borrelia burgdorferi*, *Borrelia burgdorferi* sensu stricto, Tennessee, Tennessee Valley, United States, entomologic risk, tickborne disease, vector-borne infections, bacteria, 16S rDNA, Lyme group *Borrelia*, prevalence, blacklegged ticks, Union County, surveillance, drag sampling, zoonoses

## Abstract

In 2017, we surveyed forests in the upper Tennessee Valley, Tennessee, USA. We found *Ixodes scapularis* ticks established in 23 of 26 counties, 4 of which had *Borrelia burgdorferi* sensu stricto–infected ticks. Public health officials should be vigilant for increasing Lyme disease incidence in this region.

In the United States, Lyme disease caused by tickborne bacterium *Borrelia burgdorferi* sensu stricto occurs primarily in the Northeast and upper Midwest (*1*). In eastern Tennessee, which is considered nonendemic for Lyme disease, most of the human population resides in a low-elevation swath of the Tennessee Valley bordered to the west by the Cumberland Plateau and the east by the Great Smoky Mountains. The vector of Lyme disease, the blacklegged tick *Ixodes scapularis*, was unreported in this area before 2006; in this year, uninfected adult ticks were collected from hunter-harvested deer in 8 Tennessee Valley counties (Figure 1, panel A) (*2*). This finding, plus uninfected *I. scapularis* ticks detected in Knox County in 2013, were later incorporated into the national distribution map for *I. scapularis* ticks (*3*).

**Figure 1:**
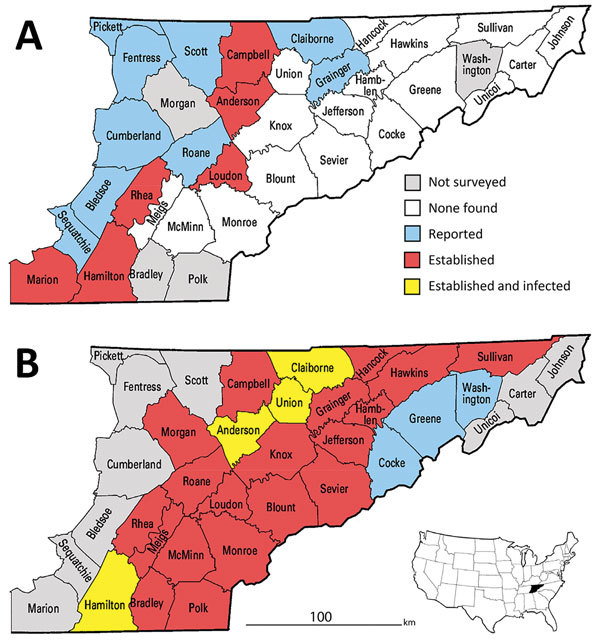
County-level distribution of *Ixodes scapularis* ticks and *Borrelia burgdorferi*–infected *I. scapularis* ticks in upper Tennessee Valley, USA, 2006 and 2017. A county was classified as having an established *I. scapularis* population if >6 *I. scapularis* adult ticks or ticks of 2 life stages were collected in that county. A county was classified as having *I. scapularis* ticks reported if 1–5 *I. scapularis* ticks of a single life stage were collected in that county. A county was classified as infected if *I. scapularis* ticks infected with *B. burgdorferi* were detected in that county. A) *I. scapularis* ticks in 2006 (*2*), determined by collecting ticks from hunter-harvested deer. B) *I. scapularis* ticks in 2017 determined by drag-cloth surveying during the peak of adult tick activity (late October–January).

During 2000–2014, human Lyme disease cases expanded southward along the eastern foothills of the Appalachian Mountains in nearby Virginia (*4*). In the winters of 2012 and 2013, *B. burgdorferi*–infected adult *I. scapularis* ticks were detected in Pulaski County, Virginia (*5*). This report of abundant infected *I. scapularis* ticks only 100 km from the Tennessee border motivated us to investigate whether *Borrelia*-infected ticks might now be present in the Tennessee Valley.

## The Study

In late 2017, we sampled host-seeking *I. scapularis* ticks at 70 forested sites in 26 low-elevation counties in the upper Tennessee Valley (Figure 1, panel B). To find tick habitats (hardwood or conifer forests <800 m in elevation) accessible for sampling (i.e., trails through public forests or margins of public roads through private forests), we reviewed Google Earth (https://www.google.com/earth/) satellite imagery. We sampled each site once during the peak of adult *I. scapularis* tick activity (late October–January). We recorded site elevation and geo-coordinates and collected host-seeking ticks using a standardized drag-cloth method; in brief, we dragged a 1-m^2^ white corduroy cloth across leaf litter and checked every 10 paces for attached ticks. We dragged cloths 30–60 minutes per site and described tick tallies as number collected per hour to correct for variations in effort per site. We did not conduct drag-cloth collections during periods of rain, strong wind, low air temperatures (<8°C), or low relative humidity (<40%).

We placed ticks in 70% ethanol, identified species using a morphologic key (*6*), and tested ticks for *Borrelia* spirochete infection by DNA extraction and quantitative multiplex real-time PCR using differential probes targeting the 16S rDNA of Lyme group *Borrelia* and relapsing fever group *Borrelia* (*7*). We then subjected a random subset of negative samples and samples positive by the 16S assay (maximum 6 samples/site) to PCR amplification of the 16S–23S rDNA intergenic spacer region (*8*) and Sanger sequencing for species-level identification.

No previous tick drag-cloth counts existed for the counties in our survey area, except for a 1,050-m transect of land in a forest in Anderson County, which we have drag-cloth sampled annually each December since 2012. To assess a trend in adult *I. scapularis* tick abundance, we applied linear regression modeling to the tick tallies from that transect of land.

**Figure 2:**
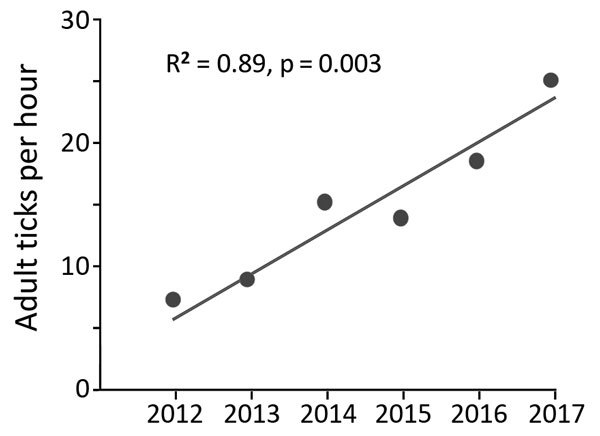
Six-year trend in adult *Ixodes scapularis* tick counts at Forest Resources Research and Education Center (36.00°N, 84.22°W; elevation 298 m), Anderson County, Tennessee, USA, 2012−2017. We collected host-seeking *I. scapularis* adult ticks by drag-cloth sampling vegetation on a 1,050-m transect of mixed hardwood forest once each December.

In late 2017, we collected 479 adult *I. scapularis* ticks from 49 of 70 sites in the upper Tennessee Valley. Two adult *Amblyomma americanum* ticks collected during the survey were excluded from analysis. We detected *I. scapularis* ticks in all 26 counties surveyed, 23 of which met the criterion used by Eisen et al. for established *I. scapularis* populations (Figure 1, panel B) (*3*). Site elevations were 210–730 m; the highest elevation at which *I. scapularis* ticks were found was 570 m. The average number of adult ticks collected per hour during drag-cloth surveys was 8.8 (range 0–48). At the Anderson County site that had been drag-cloth sampled annually, a highly significant increasing trend in *I. scapularis* ticks was evident (p = 0.003; Figure 2); the count in 2017 (24.8 ticks/hour) was 3.5× higher than that in 2012.

We tested all *I. scapularis* ticks collected (N = 479) for *Borrelia* spp. infection; 46 ticks (9.6%) from 7 sites in 4 counties (Anderson, Claiborne, Hamilton, and Union; Figure 1, panel B) tested positive for Lyme group *Borrelia* by 16S rDNA PCR screening. We tested 26 samples for the intergenic spacer region by PCR; all were positive for this sequence and identified as *B. burgdorferi* sensu stricto by sequencing. Most infected ticks came from 2 Union County sites, which had prevalences of 44% (14/32) and 78% (18/23). No ticks were found to be infected with *B. miyamotoi* or other relapsing fever group borreliae.

## Conclusions

In eastern Tennessee, public awareness and concern about ticks focuses primarily on the abundant lone star ticks (*Amblyomma americanum*) and American dog ticks (*Dermacentor variabilis*) encountered during the spring and summer. Both species can spread pathogens (*9*), but neither are vectors of *B. burgdorferi* spirochetes. Immature *I. scapularis* ticks are similarly active in the summer, but in southern states, these ticks typically avoid host-seeking above leaf litter and are rarely seen on humans or drag-cloths (*10*). For this reason, assessment of *I. scapularis* distribution in southern states is best achieved by acquiring adult life-stage ticks during cool season drag-cloth surveys (as reported here) or by collecting ticks from deer harvested in the fall. Inspection of hunter-harvested deer is efficient for the detection of low-density *I. scapularis* ticks (*11*). Thus, our drag-cloth sampling for *I. scapularis* ticks in 14 counties where none were found on deer a decade ago (Figure 1, panels A, B) suggests that tick abundance in these counties has increased. This suggestion is supported by a >3-fold increase in *I. scapularis* tick counts at the Anderson County site where we have 6 consecutive years of drag-cloth counts.

This study documents emergence of *B. burgdorferi* senso stricto in tick populations in eastern Tennessee. Infected ticks were predominantly found in high-prevalence hot spots in Union County (36.39°N). Relative to Lyme disease–endemic areas in the north, *B. burgdorferi* prevalence in the study area was low (10%) and had a patchy distribution (7/49 sites had positive ticks). This distribution could reflect host barriers of *B. burgdorferi* transmission in the South (*12*), or more concerning, the hot spots in Union County might reflect the beginning of an infection surge, similar to that seen in southwestern Virginia during the past decade (*4*).

In the United States, Lyme disease is primarily a summertime disease associated with bites from nymphal *I. scapularis* ticks. In southern states, detection of *B. burgdorferi* bacteria in adult ticks does not necessarily imply risk to humans; for example, *B. burgdorferi* cycles in *I. scapularis* populations on the Outer Banks of North Carolina, yet nymphs in that area cannot be collected on drag-cloths and no locally acquired cases of Lyme disease have been reported (*13*). In contrast, infected nymphs have been found on drag-cloths from surveys in Virginia, where Lyme disease incidence has spiked (*14*). We speculate that *Borrelia*-infected *I. scapularis* populations emerging in southwestern Virginia include immigrant ticks from the North, with some nymphs in these populations exhibiting host-seeking behaviors that lead to contact with humans. A similar invasion process might be under way in eastern Tennessee; the surveillance data reported here provide a baseline for investigating this possibility. Health officials and practitioners need to be vigilant for increasing Lyme disease incidence in Tennessee.
